# Robustness to High Temperatures of Al_2_O_3_-Coated CsPbBr_3_ Nanocrystal Thin Films with High-Photoluminescence Quantum Yield for Light Emission

**DOI:** 10.1021/acsanm.0c01525

**Published:** 2020-07-16

**Authors:** Milan Palei, Muhammad Imran, Giulia Biffi, Liberato Manna, Francesco Di Stasio, Roman Krahne

**Affiliations:** † Istituto Italiano di Tecnologia, Via Morego 30, 16163 Genova, Italy; ‡ Dipartimento di Chimica e Chimica Industriale, Università degli Studi di Genova, Via Dodecaneso, 31, 16146 Genova, Italy

**Keywords:** lead-halide perovskite, nanocrystals, quantum dots, photoluminescence, stability, amplified spontaneous emission, high temperature, atomic layer deposition, oxides

## Abstract

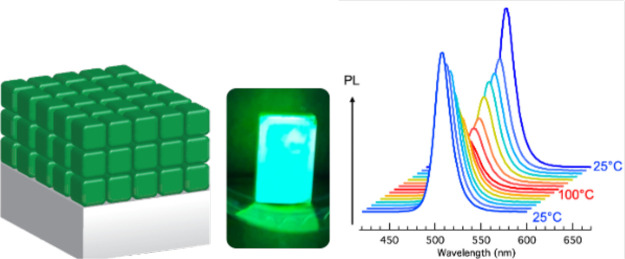

Lead-halide perovskite nanocrystals are a promising material in optical devices due to their high photoluminescence (PL) quantum yield, excellent color purity, and low stimulated emission threshold. However, one problem is the stability of the nanocrystal films under different environmental conditions and under high temperatures. The latter is particularly relevant for device fabrication if further processes that require elevated temperatures are needed after the deposition of the nanocrystal film. In this work, we study the impact of a thin oxide layer of Al_2_O_3_ on the light emission properties of thin nanocrystal films. We find that nanocrystals passivated with quaternary ammonium bromide ligands maintain their advantageous optical properties in alumina-coated films and do not suffer from degradation at temperatures up to 100 °C. This is manifested by conservation of the PL peak position and line width, PL decay dynamics, and low threshold for amplified spontaneous emission. The PL remains stable for up to 100 h at a temperature of 80 °C, and the ASE intensity decreases by less than 30% under constant pumping at high fluence for 1 h. Our approach outlines that the combination of tailored surface chemistry with additional protective coating of the nanocrystal film is a feasible approach to obtain stable emission at elevated temperatures and under extended operational time scales.

## Introduction

Perovskite nanocrystals (NCs)^1−5^ currently show record performance in various optoelectronic devices. Light-emitting diodes already present external quantum efficiencies exceeding 16% in the green spectral region,^6,7^ and very recently amplified spontaneous emission under continuous-wave excitation has been reported.^8^ Such optoelectronic performance has been reached after only a few years of intense research following the seminal work from Protesescu et al.^9^ Yet, a major hindrance of perovskite NCs is their limited stability,^10−13^ which leads to relatively short operational lifetimes of the respective devices notwithstanding the performance.^14^ In order to tackle this stability issue,^1^ various computational studies have identified the NC surface as the main culprit.^15−18^ In addition, experimental studies on NCs synthesized via different methods^19−21^ have pointed out that the nature of the surface ligands plays a major role in the material stability. In this context, postsynthetic treatments are an interesting venue to increase the stability of perovskite NCs as well as to improve photoluminescence quantum yield (PLQY);^22^ such methods include ligand exchange procedures,^23^ amines addition,^24^ cross-linking,^25^ MnCl_2_ doping,^26^ potassium incorporation,^27^ and more. Atomic layer deposition (ALD) of a thin Al_2_O_3_ layer on NC films provides an additional pathway to improve stability and performance of thin films of NCs.^28−33^

One important issue is the stability of the active layers under heating^34^ and at elevated temperatures in the range up to 100 °C. Such temperatures can occur during possible processing steps that follow the NC deposition, as, for example, resist baking in patterning by lithography or thermal evaporation of additional layers, but also during operation under high current density in LEDs or under harsh environmental conditions. The deposition of a thin oxide film, for example Al_2_O_3_, by ALD can be employed to improve the robustness of NC films to environmental conditions.^28^

**Figure 1:**
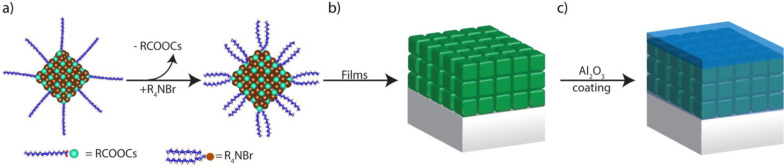
(a) CsPbBr_3_ NCs passivated with Cs-oleate ligands are obtained from synthesis and a ligand exchange to DDAB is performed in solution. (b) Thin films of NCs are fabricated by spin coating the ligand-exchanged solutions on glass substrates. (c) The films are coated with a thin Al_2_O_3_ layer via atomic layer deposition.

In this work, we investigate the impact of coating CsPbBr_3_ NC films with a thin Al_2_O_3_ layer via ALD. We use CsPbBr_3_ NCs passivated by didodecyl dimethylammonium bromide (DDAB) ligands (Figure 1) that have demonstrated excellent stability in solution and in films under ambient conditions.^23^ We obtain a PLQY of 75% from NC films prepared by spin-coating the colloidal solutions on soda lime glass substrates that is maintained after the deposition of the Al_2_O_3_ layer. We study the temperature stability of bare and alumina-coated NC films in heating and cooling cycles and find that the optical properties of Al_2_O_3_-coated films are not altered after a heating cycle up to 100 °C. Exceeding this temperature leads to irreversible reduction in emission intensity, which is analyzed in terms of PL decay lifetimes. With pulsed laser excitation, we evaluate the amplified spontaneous emission threshold and obtain a value of around 60 μJ/cm^2^ for both bare and alumina coated NC films. The PL of the films is stable for up to hundreds of hours at a temperature of 80 °C, and the ASE manifests only a moderate decrease in performance for optical pumping up to more than one hour. Such stability is of great advantage for reliable device operation and for the processing of devices that occurs after the deposition of the NC film.

## Results and Discussion

**Figure 2:**
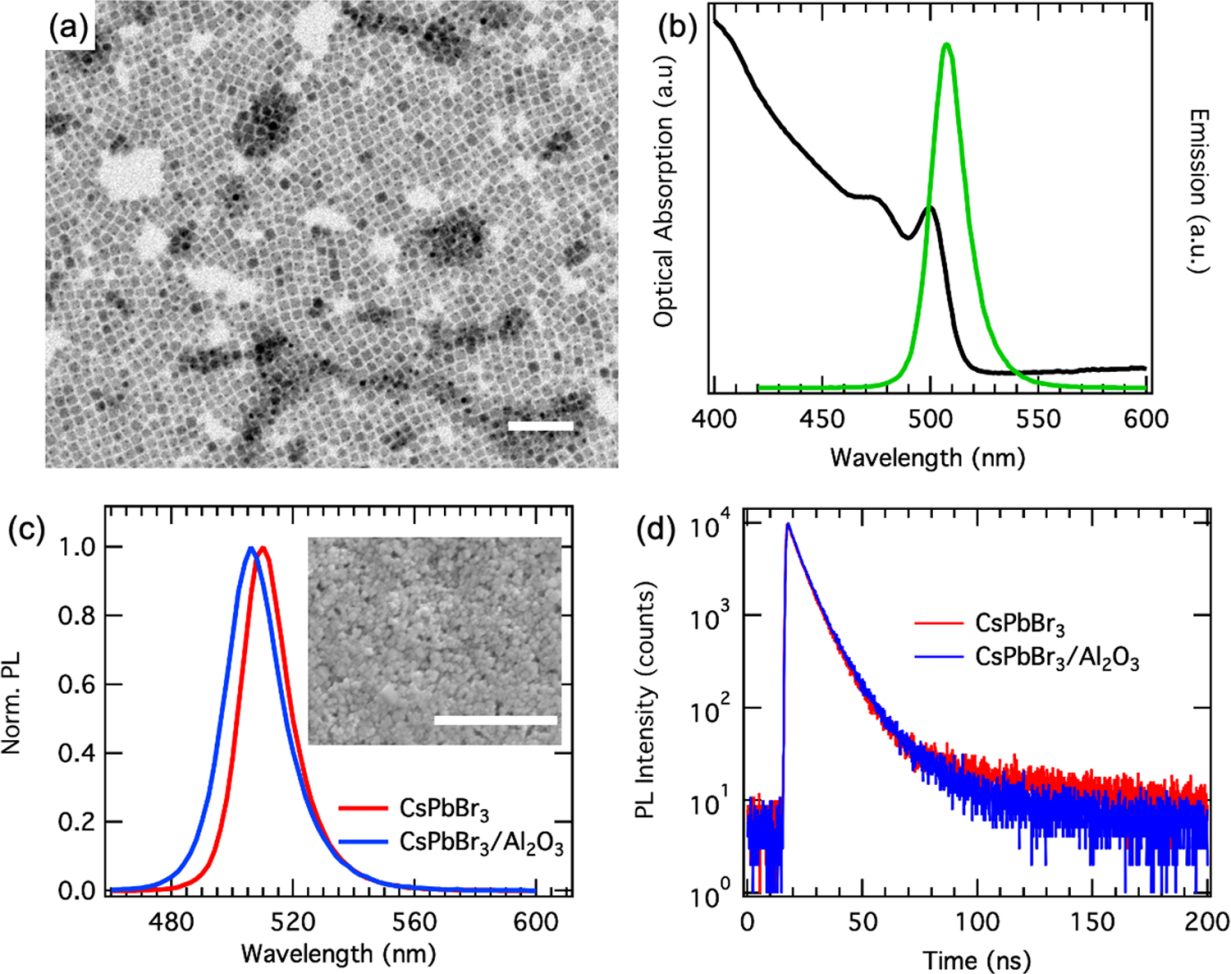
Bare and Al_2_O_3_-overcoated CsPbBr_3_ DDAB-capped NC films. (a) Transmission electron microscopy image of the CsPbBr_3_ NCs; scale bar is 100 nm. (b) Optical Absorption and PL spectra recorded from the NCs in solution. (c) Normalized PL emission of spin-coated CsPbBr_3_ NC films, bare (red) and overcoated with a 13 nm thick layer of Al_2_O_3_ (blue). The inset shows an SEM image of the NC film demonstrating the homogeneity of the deposition. Scale bar is 1 μm. (d) PL decay traces of the bare and Al_2_O_3_-coated films.

Cs-oleate-capped CsPbBr_3_ NCs were prepared following our previously reported secondary amine-based synthesis procedure (see Materials and Methods for details).^35^ A postsynthesis ligand exchange was used to displace the native Cs-oleate ligands with DDAB^23^ (Figure 1). Contrary to the more commonly used primary alkyl ammonium or alkyl carboxylate ions, which can lose or acquire a proton, hence becoming charge neutral and detaching from the surface of the NCs, quaternary ammonium ions are more stable surface-passivating agents. The surface passivation with DDAB delivers NCs with near-unity PLQY in the solution phase without affecting the overall morphology and structural properties of the NCs^23^ and therefore significantly increases the brightness of the NCs with respect to their Cs-oleate counterparts. A transmission electron microscopy image and absorption/emission spectra recorded from DDAB-passivated NCs in solution are displayed in Figure 2a,b. The NC films were fabricated via spin-coating of concentrated solutions (5 mg/mL) on a soda-lime glass substrate (2000 rpm for 60 s), and a film thickness of 50 ± 5 nm was measured via atomic force microscopy (Nanosurf, noncontact mode). The Al_2_O_3_-coated films were treated with 200 cycles of thermal ALD that resulted in an alumina layer thickness of 13 nm. The excellent film homogeneity can be appreciated in the confocal fluorescence microscopy image of the NC film in Figure S1a. Because of their brighter emission both in solution and in films, we focus on the following on the DDAB-passivated samples for the temperature and stability characterization.

Figure 2c shows the emission spectra of the pristine and Al_2_O_3_-coated NC films together with the PL decay traces. The PL peak position of the Al_2_O_3_-coated film is slightly blue-shifted with respect to that of the bare NC film, which most likely is due to dielectric effects.^36,37^ The emission intensity is not affected by the alumina overcoating, and we obtained a PLQY of 75 ± 8% at room temperature from both bare and Al_2_O_3_-coated films. The PL decay of the bare and Al_2_O_3_-coated films is very similar, with average PL decay times of τ = 7.5 ns for bare CsPbBr_3_ films and τ = 8 ns for Al_2_O_3_-coated CsPbBr_3_ films. The minor change in average lifetime is mostly attributed to a small change in the dielectric environment caused by the alumina infilling of the voids.^37^ We conclude that the photophysics of the NC films is not affected by the coating with the Al_2_O_3_ layer. X-ray diffraction spectra recorded of bare and Al_2_O_3_-coated films confirm the structural stability (Figure S1b). Next, we investigate the PL properties of the films at temperatures up to 100 and 150 °C and how such heating affects their emission once the film is cooled back to room temperature.

**Figure 3:**
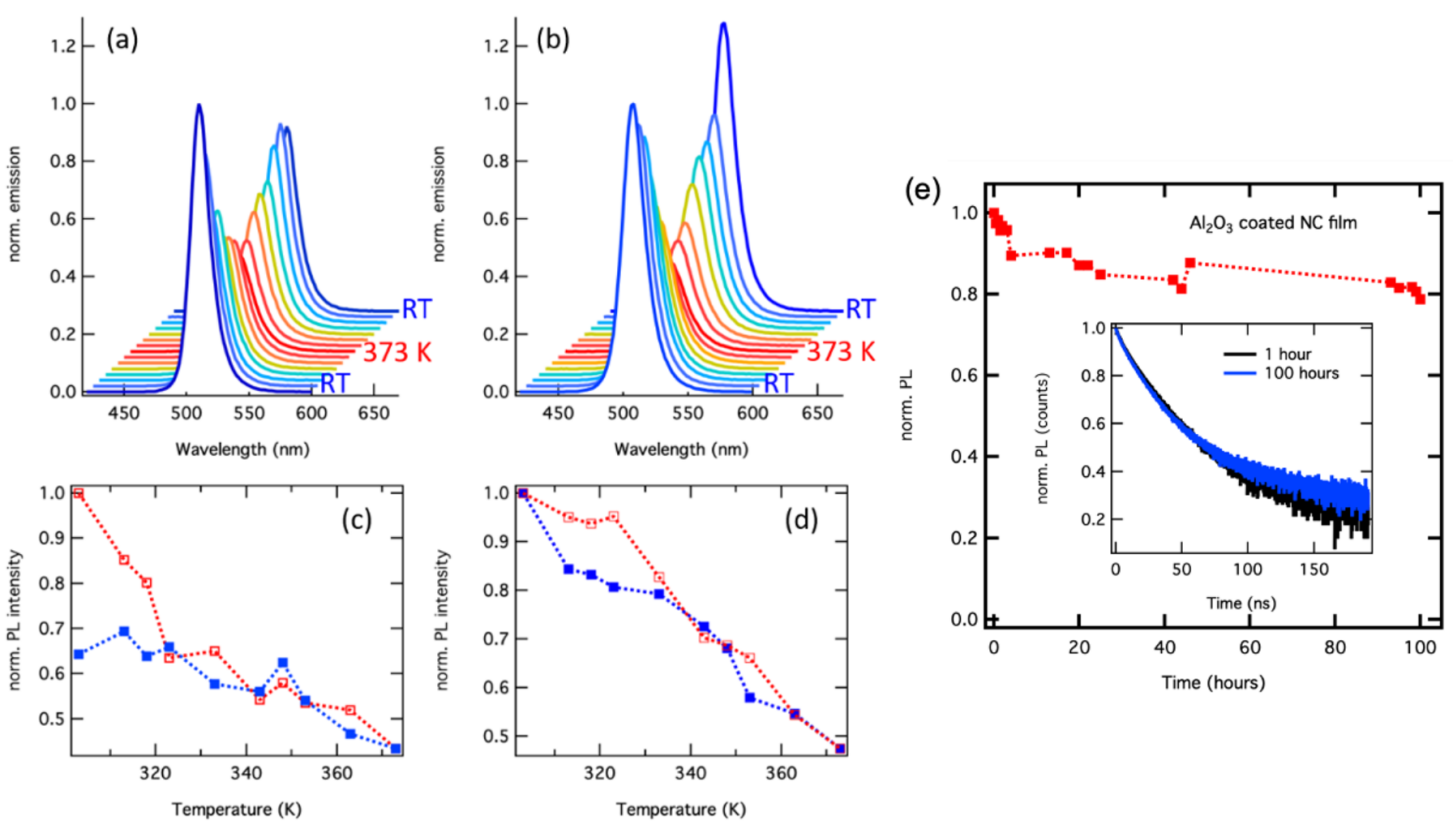
PL emission during heating and cooling cycles of bare and Al_2_O_3_-coated CsPbBr_3_ NC films. (a,b) PL spectra recorded during heating the bare (a) and Al_2_O_3_-coated (b) films up 100 °C (373 K) and cooling back to room temperature (RT). (c,d) Normalized PL intensity obtained by integrating the area under the PL peak for bare (c) and Al_2_O_3_-coated (d) films. The PLQY of the bare film drops from 75% to 44% after one heating/cooling cycle, while that of the Al_2_O_3_-coated film fully recovers. Here, unity in PL intensity corresponds to PLQY of 75%. (e) PL intensity at 80 °C (353 K) recorded over time. The emission intensity remains above 80% for up to 100 h, and the PL decay traces recorded after 1 and 100 h (plotted in the inset) do not show any significant changes, confirming the stability of the optical properties.

Figure 3a,b shows the emission spectra for heating/cooling cycles up to 100 °C (373 K) of bare and Al_2_O_3_-coated films. With increasing temperature, the PL signal decreases, demonstrating only minor changes in shape and line width. The Al_2_O_3_-coated film recovers the full PL intensity (PLQY of 75%) upon cooling back to room temperature, while the PLQY of the bare NC film is reduced after one heating/cooling cycle to 44 ± 4%. Humidity, ambient air, and temperature are known to influence the emission properties of perovskite NC films.^38−43^ The temperature cycling in Figure 3b,d demonstrates that the film coating with the alumina layer provides a sufficient protection that suppresses NC degradation for temperatures up to 100 °C. We attribute the main mechanism for the increased stability to the blocking of the desorption of the ligands that occurs at temperatures above 80 °C^38^ that preserves the efficient surface passivation of the NCs. Similar data is shown for Cs-oleate-coated films in Figure S2, however in this case the PL intensity was already significantly reduced by the Al_2_O_3_ coating itself, and furthermore the alumina-coated films did not recover their full PL intensity after being cooled down to room temperature. Another important point is the stability over time at such elevated temperatures, which is reported in Figure 3e, where the PL intensity versus time under operation at a temperature of 80 °C is plotted for up to 100 h. We find a small decrease in PL intensity in the first few hours, and then the signal stabilizes at around 80% of its original intensity.

**Figure 4:**
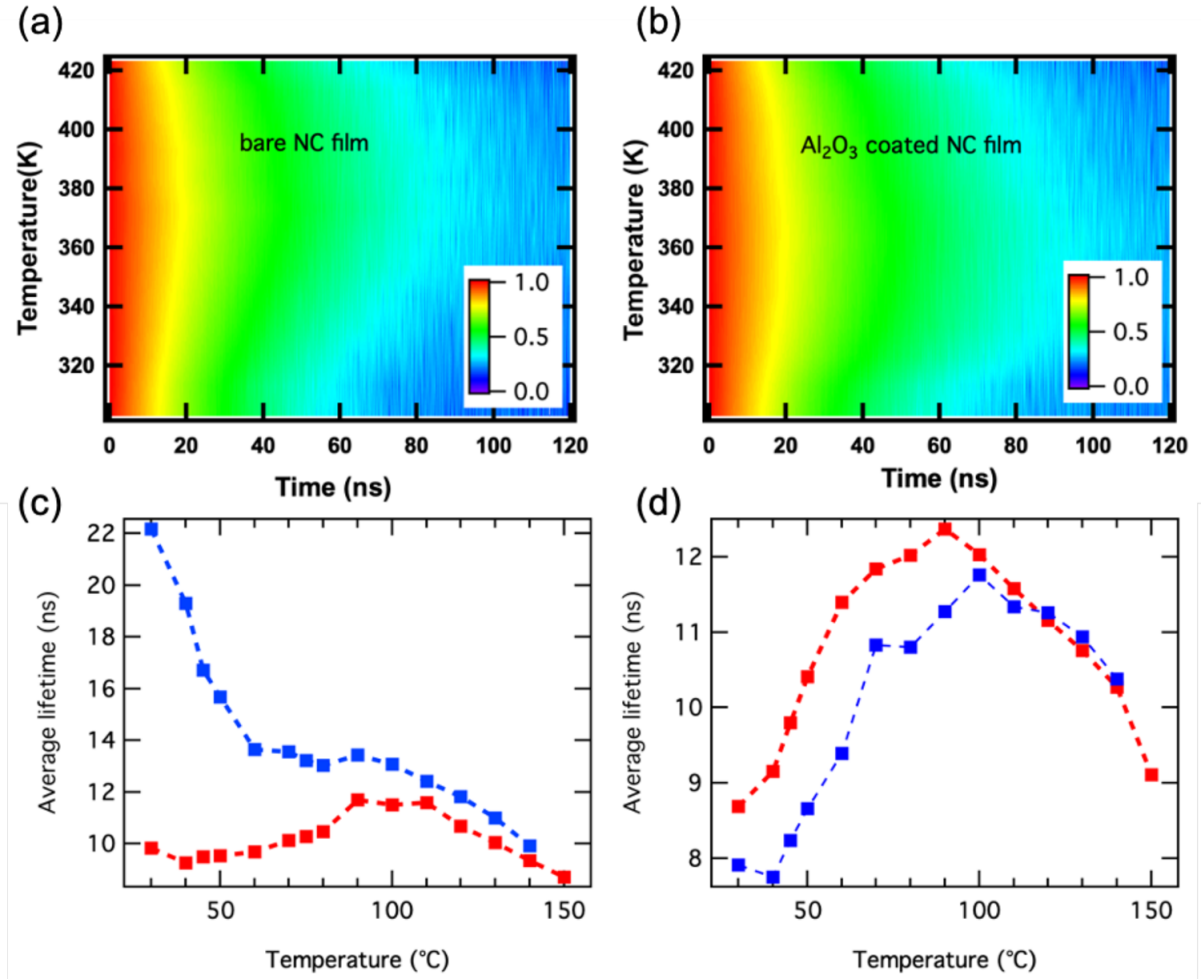
(a,b) Contour plots of the PL decay traces versus temperature of bare and Al_2_O_3_-coated films in a heating cycle up to 150 °C (423 K), and the related average PL life times (c,d) obtained from fitting with three exponentials for both heating (red) and cooling (blue) cycles.

We have also tested the robustness of the NC film emission to temperatures exceeding 100 °C and found that the PL intensity is significantly reduced after heating up to 150 °C (423 K) for both bare and Al_2_O_3_-coated films, that is, it recovered only 60% and 50% of its original value, respectively, as shown in Figure S3. We analyze the PL decay dynamics to gain deeper insight into this loss of emission intensity. The PL decay traces recorded over temperature displayed in Figure 4a,b show a maximum in PL lifetime around 100 °C (373 K). The decrease of the PL lifetime at temperatures higher than 100 °C can be attributed to fast nonradiative decay channels associated with heat-induced permanent defects, since in that case the PL is drastically and irreversibly reduced when the sample is cooled down to RT. This interpretation is supported by a shift in the amplitudes related to the two dominating decay channels, from τ_2_ (slow) to τ_1_ (fast), which is in line with an increase of the commonly faster nonradiative recombination related to defects. This effect is much more pronounced for the bare NC films (Figure 4d), for which the desorption of ligands at 150 °C is highly probable,^38^ leaving nonpassivated surface regions behind. For the Al_2_O_3_-overcoated films, the desorption is hindered by the coating layer, but the reduction of the PLQY of the film to 52% after cooling back to RT indicates that damage to the NCs or their ligand passivation occurs. The average PL lifetime measured after cooling back to room temperature is drastically different for the bare NC films compared to the Al_2_O_3_-overcoated ones. The bare films manifest a strongly increased PL lifetime from 7.5 to 22.1 ns after heating, while the overcoated ones experience a small PL lifetime decrease from 8.7 to 7.9 ns (Table S1 in SI). This behavior points to the formation of deep, long-lived traps in the bare films caused by the heating, while for the alumina-coated films no such drastic effect occurred, and only the already present fast nonradiative decay channel gained in weight. This interpretation is corroborated by PL lifetimes associated with nonradiative defects, which are typically shorter (around 2–5 ns) than those of the radiative channels (around 10–15 ns).^44^

**Figure 5:**
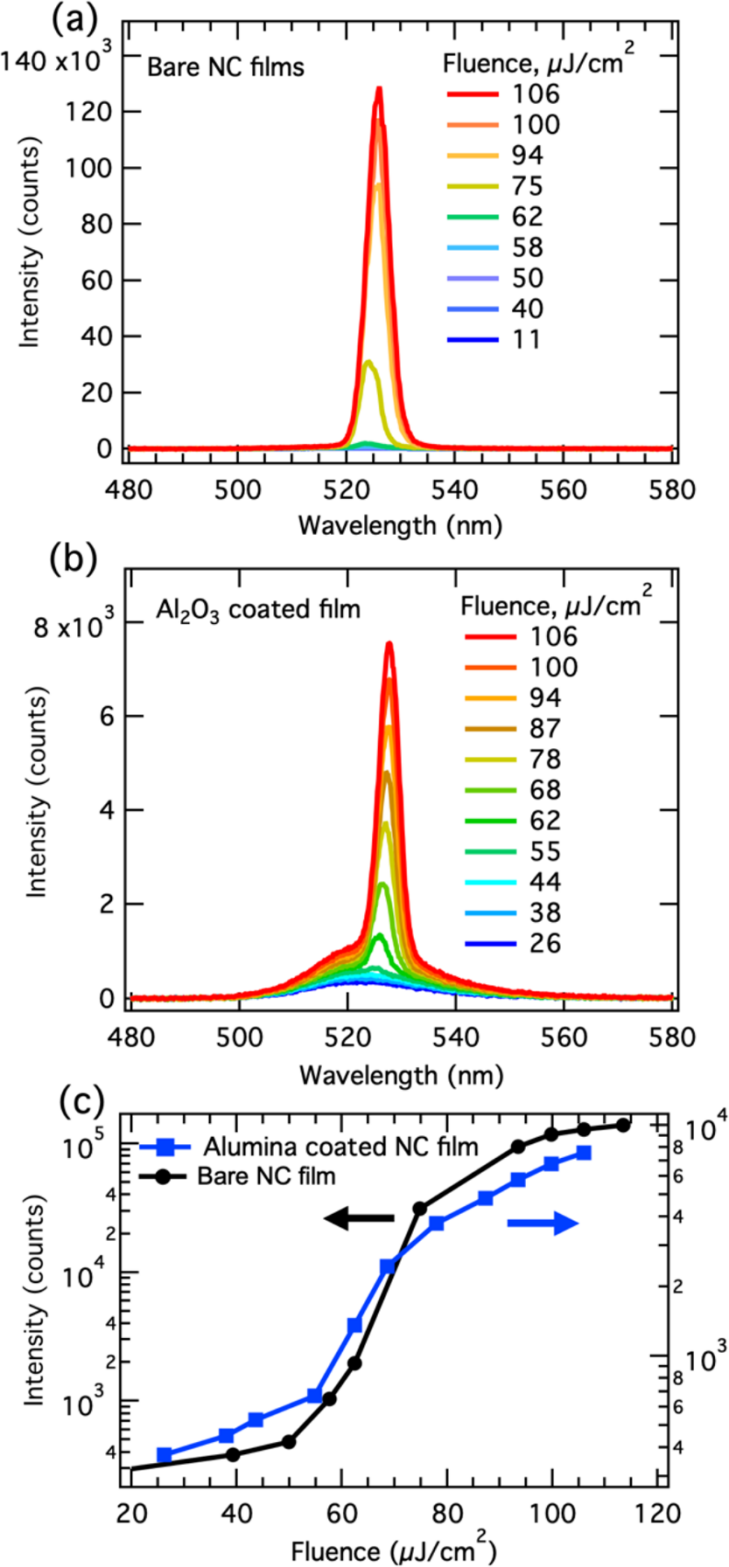
(a-b) Amplified spontaneous emission under femtosecond-pulsed laser at 405 nm at a frequency of 1 kHz for bare (a) and Al_2_O_3_-coated (b) films. The emission is recorded at grazing angles to the sample surface and some selected ASE spectra for different pump fluence are shown. (c) Emission intensity versus pump fluence for the full data set, showing an ASE threshold around 60 μJ/cm^2^.

**Figure 6:**
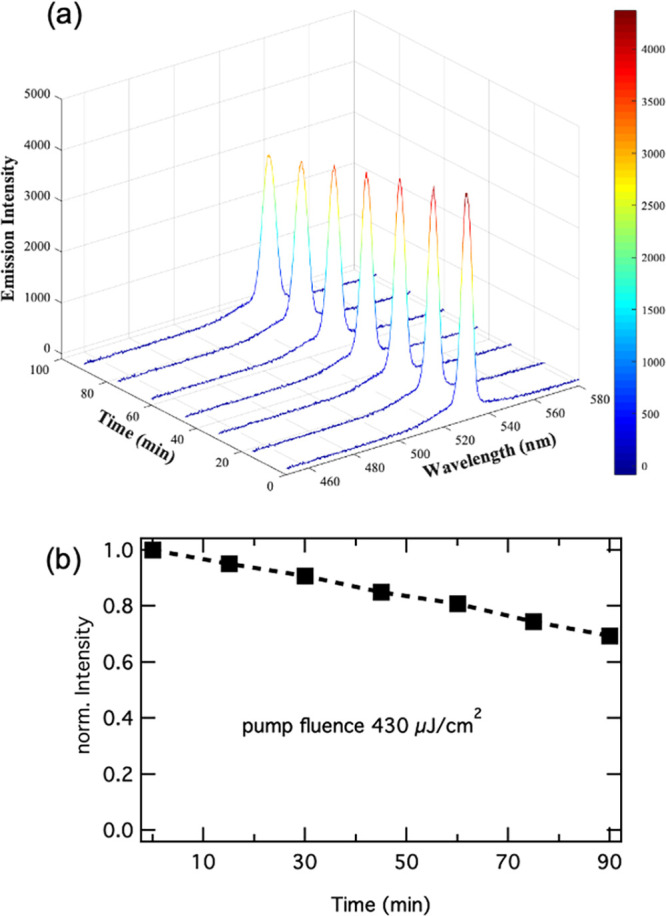
Emission spectra (a) and ASE peak intensity (b) of an Al_2_O_3_ coated NC film recorded over time under constant optical pumping with a fluence of 430 μJ/cm^2^.

CsPbBr_3_ NC films are a very interesting material for amplified spontaneous emission (ASE)^45−50^ and lasing.^51,52^ Therefore, we investigated what effects the film protection by the alumina coating has on these properties. In Figure 5a,b, we report the ASE spectra of bare and Al_2_O_3_-coated DDAB-capped CsPbBr_3_ NC films (from a different synthetic batch as in Figures 2–4). In both cases, a clear ASE peak is observed with full width at half-maximum (fwhm) of ∼4.5 nm for pump fluences exceeding 60 μJ/cm^2^. Thus, the ASE threshold is not affected by the alumina coating of the NC film. Furthermore, the threshold of 60 μJ/cm^2^ under femtosecond-excitation is comparable to other reports^53^ and roughly 1 order of magnitude higher compared to recently published results obtained from triple cation NCs that were engineered to optimize optical gain.^54^ ASE data of Cs-oleate-capped NC films are reported in Figure S4 and show comparable threshold values. The ASE peak is centered at 527 nm and therefore slightly red-shifted compared to the photoluminescence (PL) peak, since ASE arises in a spectral range where the optical losses are minimized, that is, in the Urbach tail where self-absorption is reduced. The PL peak, measured in the direction normal to the substrate, is centered at 510 nm, consistent with our previous reports,^43^ while the PL peak recorded in the ASE configuration at grazing angles (see Materials and Methods) is red-shifted to 522 nm. Such red-shift can be attributed to self-absorption caused by the long trajectory of the emitted light within the NC film. Stability tests of the ASE of the Al_2_O_3_-coated NC films over time at constant fluence of 430 μJ/cm^2^, thus around a factor of 7 above the threshold, are shown in Figure 6 and revealed a decrease in ASE intensity of only 30% after 90 min (corresponding to 5.4 × 10^6^ laser pulses). For comparison, the PL intensity of CsPbBr_3_ NCs can show a decrease of 50% (or above) under constant optical excitation,^12^ while for PbBr_2_-treated CsPbBr_3_ NC films under femtosecond-optical pumping (at a fluence of 1.5 times the threshold value) the ASE demonstrated a similar decrease after 5 × 10^6^ laser pulses.^54^ De Giorgi et al.^46^ estimated that ASE stability under nanosecond-excitation can be 4 orders of magnitude lower than that for femtosecond-pumping. In fact, the authors report a decrease of ASE intensity >50% after 3500 laser pulses employing nanosecond-excitation. Such results indicate that improvement in emission stability are required to achieve long operational lifetime, in particular in the pumping regime with longer laser pulses.

**Figure 7:**
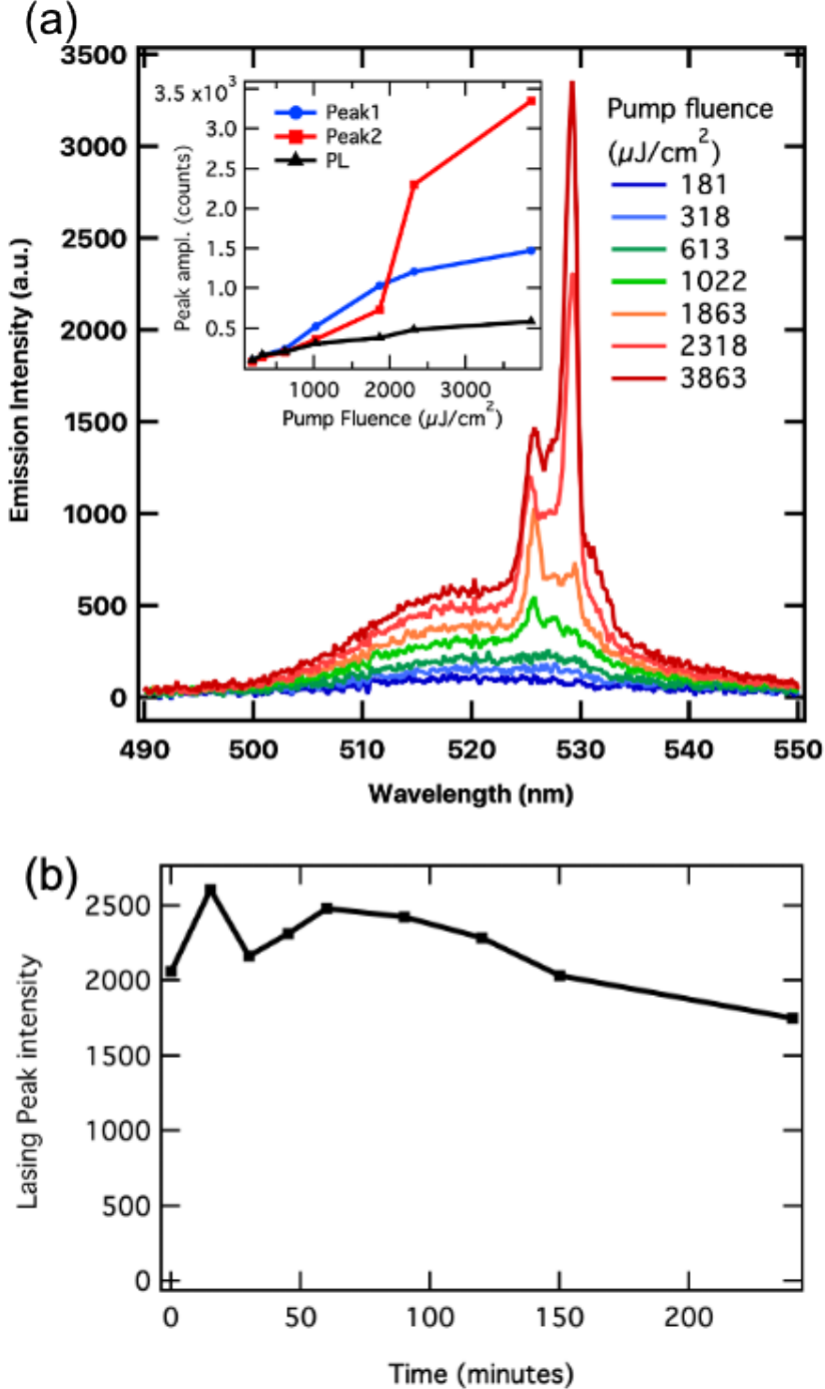
(a) Distributed feedback lasing from DDAB-capped CsPbBr_3_ NC films deposited on a silica substrate with a linear grating with 310 nm periodicity. Emission spectra for different excitation fluence show that above a threshold of 1 mJ/cm^2^ lasing peaks appear. The inset shows the PL amplitude and that of the two lasing peaks (Peak1 @ 526 nm; Peak2 @ 529 nm) versus excitation fluence. (b) Stability of the DFB laser device over time under constant pumping.

Motivated by the good stability of our NC films under high fluence pumping, we fabricated a distributed feedback (DFB) laser^55−57^ and plot the emission spectra in Figure 7a. We obtained lasing with a grating periodicity *d* = 310 nm under a detection angle of 10° with respect to the surface normal with a lasing threshold of 1 mJ/cm^2^. At the threshold, multiple peaks can be observed, markedly at 526 and 529 nm. At higher pumping fluence, the peak at 529 nm takes over and dominates the spectrum with a narrow emission line width of ∼1.6 nm. The observed mode spacing (Δλ ∼ 2.5 nm) between the two lasing peaks could result from an additional outcoupling mechanism. In fact, DFB laser emission in the direction perpendicular to the lattice plane is obtained by a second order grating that acts as a loss channel.^58^ The emission was stable over time under lasing operation at a constant pumping fluence of 2.7 mJ/cm^2^ (where a single laser peak was observed), and the peak intensity did not decrease significantly for 2.5 h (Figure 7b).

## Conclusions

We have demonstrated that the coating of CsPbBr_3_ NC films with a thin Al_2_O_3_ layer improved the robustness of the optical properties to elevated temperatures. Here, at temperatures up to 100 °C the film remained unaltered, demonstrated by full recovery of the high PLQY of 75% when cooling back to RT. Heating/cooling cycles to higher temperatures revealed that above 100 °C irreversible changes occur in the film that reduce the emission intensity, which is much more pronounced in bare NC films compared to the alumina-coated ones. Furthermore, also the threshold of ASE was unaffected by the alumina coating. The robustness of NC films up to 100 °C is highly favorable for technological applications, as many processing steps in device technology such as optical or electron-beam lithography and metal deposition rely on temperatures in this range. Also, toward the stability of optoelectronic devices under operation, heating of the active material can occur and there such robustness is paramount for stable performance. Therefore, we can foresee the exploitation of our processing technique in the stabilization of light-emitting diodes or solar cells.

## Materials and Methods

### Chemicals

Lead acetate trihydrate ((PbAc_2_·3H_2_O), 99.99%), cesium carbonate (Cs_2_CO_3_, reagent Plus, 99%), benzoyl bromide (C_6_H_5_COBr, 97%), ethyl acetate (98.8%), toluene (anhydrous, 99.5%), didodecyldimethylammonium bromide (DDDMAB), oleylamine (70%), octadecene (ODE, technical grade, 90%), and oleic acid (OA, 90%) were purchased from Sigma-Aldrich. Didodecylamine (DDDAm, 97%) was purchased from TCI. All chemicals were used without any further purification.

### Synthesis of Cs-Oleate-Capped CsPbBr_3_ NCs

Cs-oleate-capped CsPbBr_3_ NCs were synthesized following our previously reported secondary amine-based synthesis approach.^35^ Briefly, lead(II) acetate trihydrate (76 mg), cesium carbonate (16 mg), and octadecene (10 mL) were combined in a 25 mL three-neck flask equipped with a thermocouple and a magnetic stirrer. The reaction mixture was degassed for 5 min at room temperature and then for 1 h at 115 °C. Then, a ligand mixture containing oleic acid (1.5 mL, previously degassed for an hour at 120 °C and stored in a glovebox) and didodecylamine (1.25 mmol, 443 mg) dissolved in 1 mL of anhydrous toluene was rapidly injected under nitrogen. After the complete dissolution of the metal precursors, the temperature was decreased to 70 °C and a solution of benzoyl bromide (50 μL) in anhydrous toluene (500 μL) was swiftly injected. After 60 s, the reaction mixture was cooled down by using a water bath and was directly used for ligand exchange reactions.

### Ligand Exchange Reactions

DDAB-capped CsPbBr_3_ NCs were prepared following previously reported ligand exchange strategy.^23^ All ligand exchange reactions were performed under air. Briefly, the crude reaction mixture containing the CsPbBr_3_ NCs (3 mL) was treated with an anhydrous toluene solution containing the DDAB salt (2 mL, 0.025 M) and the mixture was vigorously stirred for 1 min. Subsequently, the NCs were precipitated by the addition of 15 mL of ethyl acetate followed by centrifugation at 6000 rpm for 10 min. A second cycle of ligand exchange was carried out by redispersing the NCs in a toluene solution containing the DDAB salt (1 mL, 2 mM) and washing the NC dispersion with 6 mL of ethyl acetate and redispersion in toluene.

### Transmission Electron Microscopy (TEM)

Bright-field TEM images were acquired on samples prepared by drop-casting diluted colloidal solutions on carbon film-coated 200 mesh copper grids, using a JEOL-1100 microscope operating at an acceleration voltage of 100 kV.

### Film Preparation and Characterization

CsPbBr_3_ NC solutions were spin-coated on glass substrate at 2000 rpm for 1 min, which resulted in a film thickness of 50 ± 5 nm.

### Atomic Layer Deposition

Atomic layer deposition was carried out in a Flexal ALD system from Oxford Instruments by using a thermal recipe with a stage temperature of 80 °C. Trimethyl aluminate (TMA) and H_2_O were used as precursors. Before starting the alumina deposition, a preheating step of 300 s was performed. Each ALD cycle consisted of an H_2_O/purge/TMA/purge with a pulse duration of 0.12/30/0.020/10 s, respectively. The process resulted in an alumina deposition rate of 0.065 nm/cycle.

### Optical Characterization

PL measurements of the films were carried out with an Edinburgh Instruments fluorescence spectrometer (FLS920). The system included a xenon lamp with monochromator for steady-state PL excitation, a calibrated integrating sphere for PL quantum yield (PLQY) measurements and a time-correlated single-photon-counting unit coupled with a pulsed laser diode (λ= 405 nm, pulse width = 50 ps) for time-resolved PL studies.

### Heating and Cooling Experiments

High-temperature heating cooling measurements were performed inside Edinburgh fluorescence spectrometer (FLS920) with customized temperature-controlled holder from CaLCTec srl. Measurements were performed on spin-coated and ALD-coated film on glass substrate of size 1.6 × 1.3 cm^2^ in ambient atmospheric conditions by ramping the temperature from 300 to 373 K (Cycle 1) and 300 to 423 K (cycle 2) with a step of 5–10 K followed by a natural cooling cycle. PL and lifetime were measured at each of the temperature intervals both in heating and cooling cycles.

### Distributed Feedback Laser Fabrication

The lasing structures were prepared by drop-casting the CsPbBr_3_ NC solution on a fused silica grating. The grating was bought from NIL Technology and presented linear grating intrusions within the fused silica substrate in an area of 200 μm × 400 μm.

### Amplified Spontaneous Emission and Lasing Measurements

Films of CsPbBr_3_ NCs were fabricated by drop casting (with thickness of few μm) excited at a wavelength of λ = 405 nm using an amplified Ti:sapphire laser (Coherent Legend Elite seeded by a Ti:sapphire fs laser) with a 70 fs pulse (fwhm) and a repetition rate of 1 kHz. The ASE measurements were performed by focusing the excitation beam with a cylindrical lens onto the sample, thus obtaining a stripe-shaped beam profile. All ASE spectra were collected at 90° with respect to the excitation beam using an Ocean Optics HR4000 spectrometer coupled to an optical fiber. The lasing measurements were carried out using a spherical lens for focusing the beam and placing the DFB structure 80° with respect to the excitation beam (100° with respect to the collection optics).

## Supporting Information Available

Confocal fluorescence image of NC films, X-ray diffraction spectra of films before and after Al_2_O_3_ coating, PL decay fitting details and parameters, PL spectra of DDAB-capped NC films before and after heating to 150 °C, PL and ASE spectra of films consisting of Cs-oleate-capped NCs (PDF)
