# Supplementary material for: Robustness to High Temperatures of Al2O3-Coated CsPbBr3 Nanocrystal Thin Films with High-Photoluminescence Quantum Yield for Light Emission
Source: ACS Appl Nano Mater. 2020 Jul 16;3(8):8167–75. doi: 10.1021/acsanm.0c01525 (PMC8009476; doi:10.1021/acsanm.0c01525)
Supplement: Supplementary file 1 — an0c01525_si_001.pdf [file an0c01525_si_001.pdf]

Supporting Information

Robustness to High Temperatures of Al_2O_3-Coated $CsPbBr_3$ Nanocrystal Thin Films with High Photoluminescence Quantum Yield for Light Emission

Milan Palei,[1,†] Muhammad Imran,[1] Giulia Biffi,[1,2] Liberato Manna,[1] Francesco Di Stasio,[1] Roman Krahne[1*]*

[1] Istituto Italiano di Tecnologia, Via Morego 30, 16163 Genova, Italy

[2]Dipartimento di Chimica e Chimica Industriale, Università degli Studi di Genova, Via Dodecaneso, 31, 16146 Genova, Italy

AUTHOR INFORMATION

Corresponding Authors:

*Dr. Roman Krahne, email: roman.krahne@iit.it

*Dr. Francesco Di Stasio, email: francesco.distasio@iit.it

Present Addresses:

†Department of Electrical Engineering, University of Notre Dame, IN 46556, USA

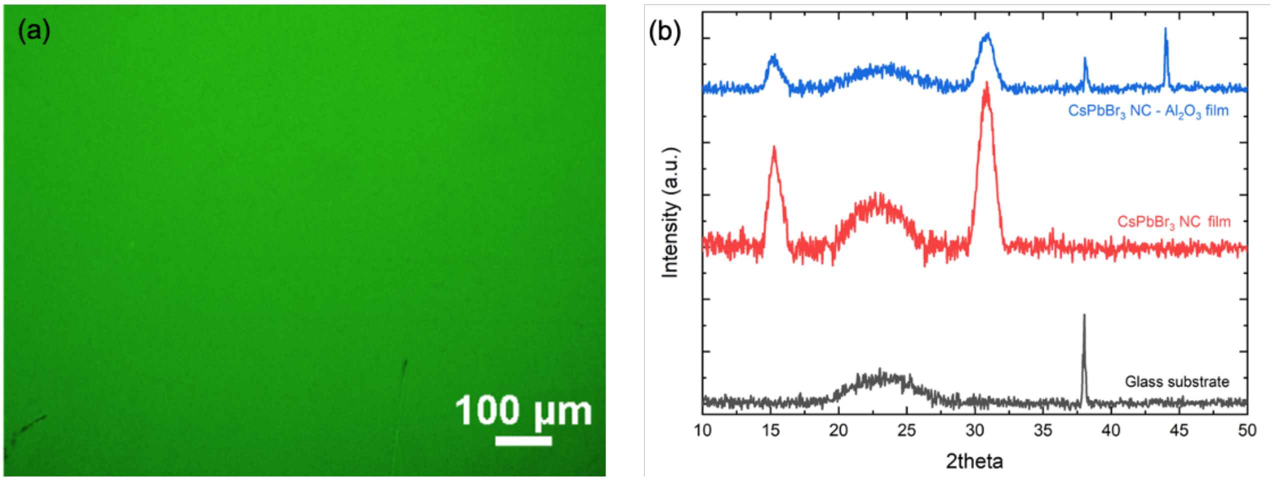

Figure S1. (a) PL confocal microscopy image of ligand-exchanged spin coated NC film that corroborates the good film homogeneity. (b) X-ray diffraction spectra recorded before and after the deposition of the Al_2O_3 layer on the DDAB-capped NC films.

PL decay fitting:

Fluorescence decay traces were fitted with three exponentially decaying function. The average lifetime (τ_{avg}) was calculated using the following relation (J.R. Lakowicz, Principles of Fluorescence spectroscopy, New York, Springer, 3rd ed. (2006)):

$$\tau_{avg} = \frac{A_1\tau_1^2 + A_2\tau_2^2 + A_3\tau_3^2}{A_1\tau_1 + A_2\tau_2 + A_3\tau_3}$$

TABLE S1. Full set of PL decay fitting parameters of data in Figure 4 c,d.

Temperature (°C)	τ1	A1	τ2	A2	τ3	A3	τ_{avg}
			Al_2O_3 coated film of DDAB capped NCs				
Heating up							
30	4.64	5735	9.58	4177	38.4	64	8.69
40	4.96	6155	10.30	4041	43.53	54	9.15
45	4.97	5195	10.42	4531	34.63	111	9.80
50	4.65	4481	10.55	5155	29.84	193	10.41
60	4.82	4403	11.34	5157	31.35	229	11.40
70	4.58	4127	11.64	5586	31.44	244	11.84
80	4.65	3990	11.67	5588	28.35	345	12.02
90	4.73	3983	12.10	5567	30.54	293	12.37
100	3.82	2745	10.36	6290	23.07	767	12.03
110	4.37	3851	11.13	5641	26.64	373	11.58

120	4.17	3810	10.73	5580	24.66	411	11.16
130	4.2	4105	10.40	5439	24.47	398	10.76
140	4.07	4499	10.32	5023	26.5	258	10.27
150	3.4	4215	8.72	5171	22.11	345	9.11
**Cooling**							
140	3.77	4010	10.01	5420	25.73	305	10.38
130	4.71	4669	11.45	4844	32.78	152	10.94
120	4.4	4049	11.12	5745	29.06	254	11.26
110	4.29	3527	10.82	5853	25.92	368	11.34
100	5.18	4433	12.20	5421	37.56	125	11.76
90	5.13	4178	11.62	5499	34.14	140	11.27
80	4.82	4047	10.94	5670	32.29	156	10.80
70	5.59	5650	11.66	4155	65.81	35	10.83
60	5.1	5457	10.3	4622	37.09	74	9.39
50	5.51	6805	10.48	3169	39.55	47	8.66
45	5.02	6178	9.50	3676	32.38	71	8.24
40	4.92	6601	9.08	3510	29.51	77	7.75
30	4.96	6917	9.92	2815	44.26	28	7.91
**Bare film of DDAB capped NCs**							
**Heating up**							
30	4.32	4589	8.53	5141	63.62	49	9.84
40	4.08	4808	8.93	4904	39.74	96	9.27
45	3.69	4401	8.99	5309	36.87	118	9.50
50	3.35	4168	8.9	5508	34.86	134	9.55
60	2.89	3800	8.71	5865	25.5	312	9.70
70	2.83	3990	9.12	5577	26.76	302	10.14
75	2.85	4075	9.00	5550	24.69	422	10.28
80	2.51	3764	8.79	5601	22.9	556	10.47
90	3.26	3974	10.58	5248	30.22	307	11.70
100	2.66	3559	9.53	5640	23.84	650	11.52
110	2.87	3642	9.86	5202	24.58	562	11.59
120	2.42	3518	8.65	5612	21.8	699	10.69
130	2.6	3859	8.73	5320	23.26	444	10.06
140	2.39	4010	7.99	5306	21.6	471	9.35
150	2.42	4779	8.01	5058	24.49	263	8.72
**Cooling**							
140	2.67	4301	8.63	5099	24.11	411	9.93
130	2.61	3591	8.89	5184	23.34	611	11.00
120	2.52	3981	9.38	5528	25.64	622	11.83
110	2.39	3501	9.15	5482	24.17	877	12.43
100	2.47	3393	9.56	5712	26.74	771	13.08
90	2.95	3968	10.23	5046	31.52	507	13.44
80	2.53	3819	8.97	5489	29.57	618	13.04
75	2.56	3932	8.82	5332	32.8	489	13.22
70	2.66	4089	8.49	5123	34.35	475	13.56
60	2.94	4690	8.33	4899	38.95	370	13.65
50	2.84	5583	7.07	4042	45.63	329	15.67
45	2.65	5589	6.32	3986	48.13	311	16.71
40	2.41	6821	5.56	3164	52.86	292	19.29
30	2.48	6928	5.12	2865	56.38	305	22.17

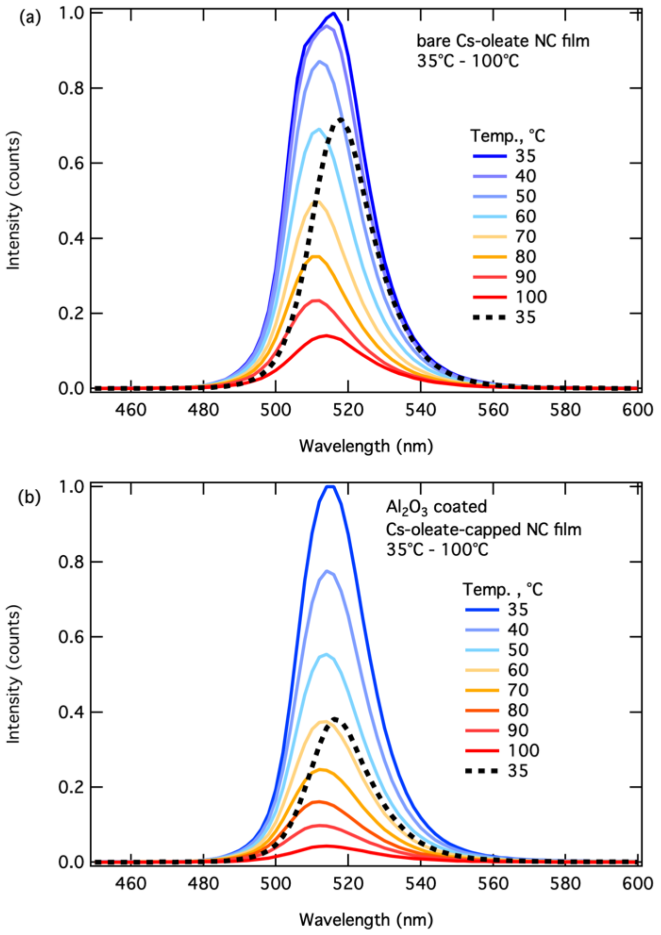

Figure S2. PL spectra of Al_2O_3 coated Cs-oleate-capped $CsPbBr_3$ NCs films at different temperatures, and after cooling back to the initial temperature (dashed line).

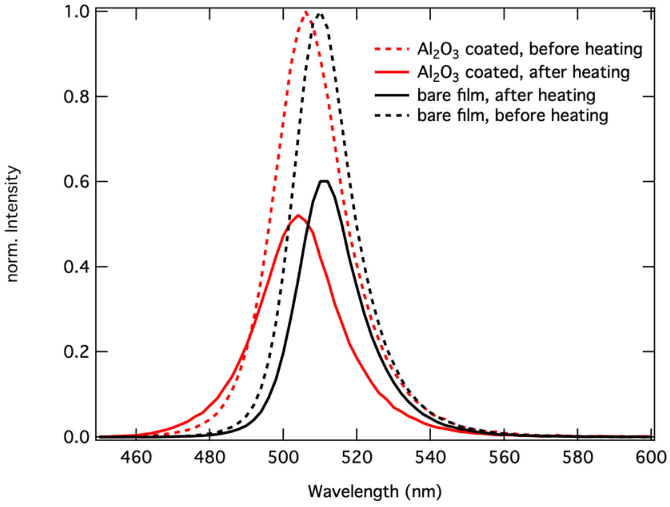

Figure S3. PL spectra recorded from bare and alumina coated films of DDAB-capped $CsPbBr_3$ NCs before and after heating to 150°C.

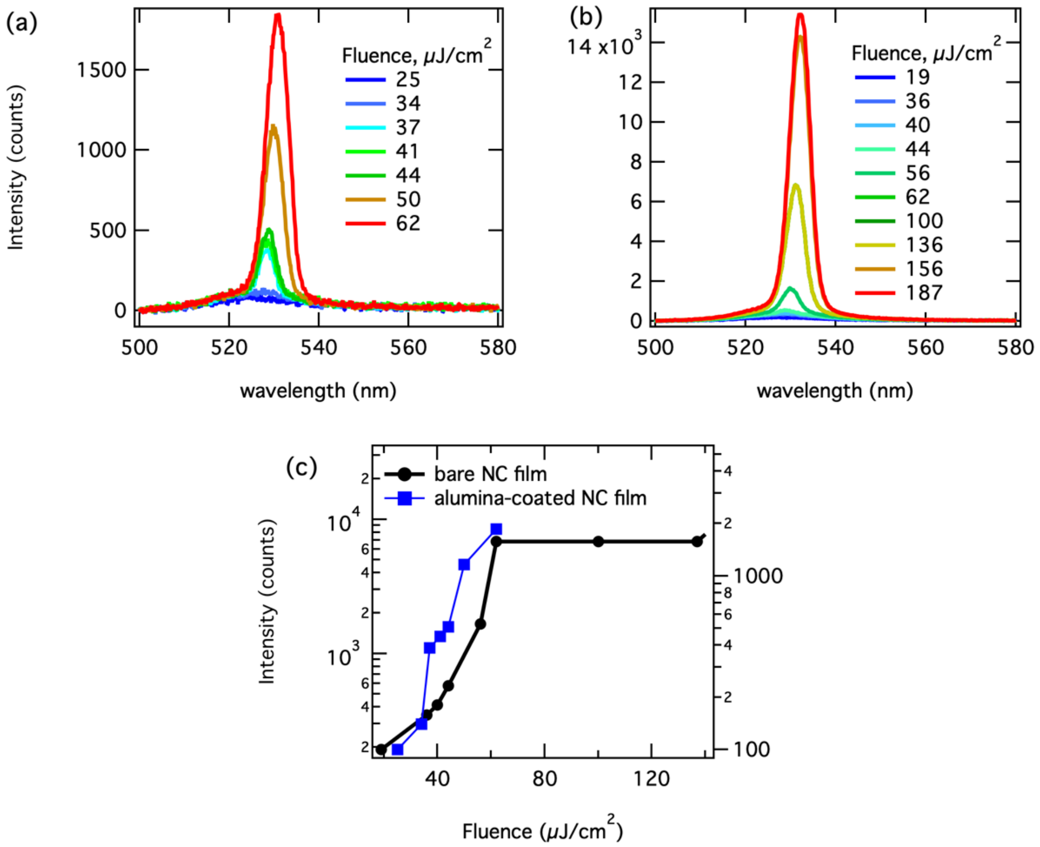

Figure S4. Amplified spontaneous emission under fs-pulsed laser at 405 nm at a frequency of 1 kHz for Al_2O_3 coated (a) and bare (b) Cs-oleate capped NC films. The emission is recorded at grazing angles to the sample surface and some selected ASE spectra for different pump fluence are shown. (c) Emission intensity versus pump fluence.

Distributed Feedback Lasing

We used a linear grating etched into a fused-silica substrate and deposited the NCs by drop-casting of the NC solution. Optical feedback in the DFB arises from the different refractive indexes from the grating (n = 1.45) and the NCs (n ~ 3). For a DFB, the cavity mode is defined by the following equation:

$$m\,\lambda = 2\,d\,\sqrt{n_{eff}^2 - \sin^2\Theta}$$

Where d is the grating periodicity, n_{eff} is the effective refractive index of the structure (NC film and grating), m is an integer, and Θ is the angle under which the emission is detected.
